# Drug-induced severe liver injury due to *Sterculia versicolor*: a case report with causality assessment from possible to probable

**DOI:** 10.7555/JBR.36.20220044

**Published:** 2022-05-28

**Authors:** Ling Zhu, Jiawei Geng, An Xiao

**Affiliations:** Department of Infectious Diseases and Hepatic Diseases, the First People's Hospital of Yunnan Province, the Affiliated Hospital of Kunming University of Science and Technology, Kunming, Yunnan 650032, China

**Keywords:** *Sterculia*, herb-induced liver injury, Roussel Uclaf Causality Assessment Method

## Abstract

*Sterculia* gum, the dry exudate of *Sterculia versicolor* and other members of the same genus, is used as a thickener and emulsifier in foods. It is generally considered safe as a food or drug, and its adverse reactions, such as *Sterculia*-induced liver injury, have never been reported. A 46-year-old woman was admitted to hospital with fatigue, nausea, abdominal distension, jaundice and a >16-fold increase in transaminase and bilirubin level. The patient had used *Sterculia* gum prior to the onset of her symptoms. Her symptoms and clinical indicators improved after treatment. The possibility of acute viral hepatitis, autoimmune hepatitis, and metabolic liver disease was excluded. After discharge from hospital, the patient had a severe liver injury again when re-exposed to *Sterculia* gum. And the Roussel Uclaf Causality Assessment Method score was updated from 5 to 7, which was consistent with probable drug-induced liver injury. This is the first report of *Sterculia*-induced liver injury. Clinicians need to be aware of the potential hepatotoxicity of *Sterculia*.

## Introduction

Drug-induced liver injury (DILI) is one of the most common and serious adverse drug reactions It can be induced by small chemical molecules, biological agents, traditional Chinese medicines, natural medicines, health products, or dietary supplements. Idiosyncratic DILI, which is more common than intrinsic DILI, can be classified into hepatocellular injury, cholestatic injury, mixed hepatocellular-cholestatic injury and vascular injury^[1]^. The severity ranges from mild, asymptomatic disturbances of liver biochemistry to fulminant hepatitis culminating in death or liver transplantation^[1]^. According to the duration of the disease, DILI is classified into either acute or chronic^[1–2]^.

*Sterculia* gum is an exudate of *Sterculia versicolor* and other members of the genus, such as *Sterculia urens*. An acid polysaccharide composed of galactose, rhamnose, and galacturonic acid, *Sterculia* gum is usually used as a thickener and emulsifier in foods, a laxative or an adhesive. It is also used as a dietary fiber supplement in its main producing areas, such as Myanmar, Vietnam, India, and other tropical and subtropical countries. Side effects of *Sterculia* gum include flatulence, abdominal cramping, and fecal impaction^[3]^. So far, there has been no report of *Sterculia*-induced liver damage caused by *Sterculia* products. Here, we report a case of severe liver injury that is most likely induced by *S. versicolor* gum. It is suggested that the safety of *Sterculia* products as dietary supplement should be evaluated by further studies.

The study was conducted according to the Declaration of Helsinki guidelines and approved by the Ethical Committee of the First People's Hospital of Yunnan Province. Written informed consent was obtained from the patient for publication of this case report.

## Case report

**Figure 1:**
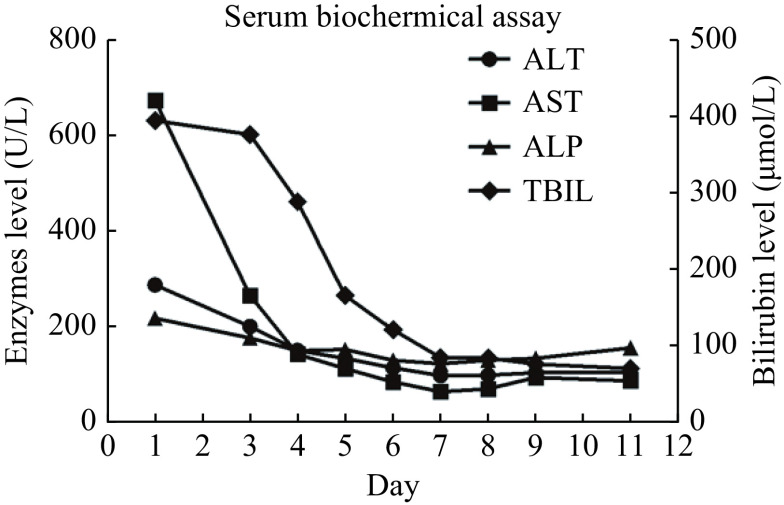
Schematic representation of trends of serum liver function tests during the clinical course and follow-up.

A 46-year-old woman was admitted to hospital on May 31, 2019, with abdominal distension, fatigue, loss of appetite, and nausea followed by jaundice after drinking herbal tea to treat her obstipation for nearly 30 days. The herbal tea was purchased from a traditional Chinese medicine pharmacy, and the similar formulations had been used before, but not for such a long period of time. There were no other known hepatotoxic medications taken in the previous month. She was diagnosed with hypothyroidism in 2014, followed by regular medication of levothyroxine, 125 μg QD, till the onset of liver injury, without history of other liver disease. She also had skin itching, but no other concomitant symptoms were found, such as joint pain, epistaxis, or gingival bleeding. Physical examination showed severe jaundice, without fever, rash, or any signs of chronic liver disease. Her blood test revealed a white blood cell count of 6.06×10^9^/L, eosinophils count of 0.05×10^9^/L, and platelet count of 340×10^9^/L. Serum biochemical assay presented the following results: albumin 34 g/L, total bilirubin (TBIL) 393.5 μmol/L, direct bilirubin (DBIL) 302.9 μmol/L, alanine transaminase (ALT) 286 IU/L, aspartate aminotransferase (AST) 672 IU/L, alkaline phosphatase (ALP) 216 IU/L, γ-glutamyl transpeptidase (γ-GT) 130 IU/L, total cholesterol (TC) 5.55 mmol/L, and triglyceride (TG) 4.72 mmol/L. Coagulation function tests showed a prothrombin time (PT) of 13.3 seconds, and an international normalized ratio (INR) of 1.01. Thyroid function was abnormal, with thyroid stimulating hormone (TSH) 0.008 mIU/L, tetraiodothyronine (T4) 189.2 nmol/L, triiodothyronine (T3) 1.23 nmol/L, free tetraiodothyronine (FT4) 23.04 pmol/L, free triiodothyronine (FT3) 3.27 pmol/L, thyroglobulin antibody (aTG) 257.5 IU/mL, thyroid peroxidase antibody (aTPO) 149.7 IU/mL. The results of the antibodies for hepatitis A, B, C, and E were all negative. Nucleic acid of Epstein-Barr virus, cytomegalovirus, and herpes simplex virus were all undetectable. Ceruloplasmin and glucose-6-phosphate dehydrogenase were normal. Anti-nuclear antibody (ANA) was positive at titer of 1:320, as was anti-neutrophellol cytoplasmic antibody (ANCA) at titer of 1:32, but all other autoimmune markers were negative, including smooth muscle antibodies. Abdominal ultrasound and magnetic resonance testing showed mild fatty liver, slightly enlarged liver and spleen, with multiple cysts within liver. Magnetic resonance cholangiopancreatography showed no calculi or obstruction dilation in the biliary tract system. Viral hepatitis was therefore excluded, and evidence of autoimmune hepatitis was insufficient. The Roussel Uclaf Causality Assessment Method (RUCAM) score was 5^[4]^, suggesting the possibility of DILI (***Table 1***). In the analysis of new R value^[5]^, our case was 8.09, suggestive of hepatocellular DILI. The severity of the liver injury was level 4. A diagnosis of hyperthyroidism and DILI was made, but the cause of DILI remained unclear. Moreover, the liver injury caused by hyperthyroidism cannot be completely ruled out. Ursodeoxycholic acid capsules (Losan Pharma GmbH, Germany), polyene phosphatidylcholine capsules (Sinofi, China), and ademetionine 1,4-butanedisulfonate (Abbott S.P.A., Italy) injection were administrated. Then methylprednisolone at a dosage of 40 mg QD was administrated for 7 days to alleviate liver inflammation after excluding viral hepatitis and tuberculosis. To rapidly lower the bilirubin level, artificial liver support system was used twice. Subsequently, her clinical indicators were improved, namely, the levels of bilirubin, ALT and AST significantly decreased at the sixth day after treatment (***Fig. 1***). The patient was discharged on June 10, while the administration of bicyclol tablet and ademetionine 1,4-butanedislfonate tablet were continued.

**Table 1:** Patient's score on the Roussel Udaf Causality Assessment Method scale at her first admission

Liver injury type	Hepatocellular	Score
Time of onset of the event	First exposure	
Time from drug intake until reaction onset	5–90 days	+2
Course of ALT after cessation of the drug/herb Percentage difference between ALT peak and ULN	Decrease more than 50% within 8 days	+3
Risk factors	No alcohol use/Age<55 years	0
Concomitant drug/herb	Concomitant drug/herb with incompatible time to onset	0
Search for alternative causes	The 6 causes of group Ⅰ ruled out	0
Previous hepatotoxicity of the drug/ herb	Reaction unknown	0
Response to unintentional re-exposure	Other situations	0
Total score	Possible	5
ALT: alanine transaminase; ULN: upper limit of normal.		

However, on the 15^th^ day after she was discharged, the same symptoms recurred without re-exposure to the same herbal tea. When admitted on June 26, the physical examination showed only severe jaundice, without fever, rash, or any other signs of chronic liver disease. Her blood tests revealed a normal white blood cell count (6.47×10^9^/L), eosinophils count (0.1×10^9^/L), and platelet count (254×10^9^/L). Serum biochemical assay showed the following results: albumin 40 g/L, TBIL 209.3 μmol/L, DBIL 154.1 μmol/L, ALT 454 IU/L, AST 367 IU/L, ALP 226 IU/L, γ-GT 240 IU/L, TC 5.68 mmol/L and TG 4.26 mmol/L. Coagulation function tests showed a PT of 13.6 seconds, and an INR of 1.04. Thyroid function was mild abnormal (T3 1.29 nmol/L), but other indicators were normal (TSH 0.826 mIU/L, T4 179.4 nmol/L, FT4 19.58 pmol/L, FT3 3.13 pmol/L). Tests for virus hepatitis all presented negative results. All the autoimmune markers, including ANA, and ANCA, were negative (***Table 2***).

**Table 2:** The patient's test results at both admissions

Items	Indicators with the normal range	Results on May 31	Results on June 26
Blood routine test	WBC ([3.5–9.5]×10^9^/L)	6.06	6.47
	EOS ([0.02–0.52]×10^9^/L)	0.05	0.1
	PLT ([125–350]×10^9^/L)	340	254
Serum biochemical assay	ALB (40–55 g/L)	34	40
	TBIL (0–21 μmol/L)	393.5	209.3
	DBIL (0–6.8 μmol/L)	302.9	154.1
	ALT (7–40 U/L)	286	454
	AST (13–35 U/L)	672	367
	ALP (50–135 U/L)	216	226
	TC (2.8–5.7 mmol/L)	5.55	5.68
	TG (0.34–1.7 mmol/L)	4.72	4.26
Coagulation function tests	PT (11–15 seconds)	13.3	13.6
	INR	1.01	1.04
Thyroid function	TSH (0.27–4.2 mIU/L)	0.008	0.826
	T4 (66–181 nmol/L)	189.2	179.4
	T3 (1.3–3.1 nmol/L)	1.23	1.29
	FT4 (12–22 pmol/L)	23.04	19.58
	FT3 (3.1–6.8 pmol/L)	3.27	3.13
	aTG (<115 IU/ml)	257.5	63.6
	aTPO (<34 IU/ml)	149.7	31.1
Autoimmune marker	ANA (Negative <1:100)	Positive 1:320	Negative <1:100
	ANCA (Negative <1:10)	Positive 1:32	Negative <1:10
WBC: white blood cell count; EOS: eosinophils count; PLT: platelet count; TBIL: total bilirubin; DBIL: direct bilirubin; ALT: alanine transaminase; AST: aspartate aminotransferase; ALP: alkaline phosphatase; TC: total cholesterol; TG: triglyceride; PT: prothrombin time; INR: international normalized ratio; TSH: thyroid stimulating hormone; T4: tetraiodothyronine; T3: triiodothyronine; FT4: free tetraiodothyronine; FT3: free triiodothyronine; aTG: thyroglobulin antibody; aTPO: thyroid peroxidase antibody; ANA: antinuclear antibodies; ACAN: anti-neutrophil cytoplasmic antibodies.			

**Figure 2:**
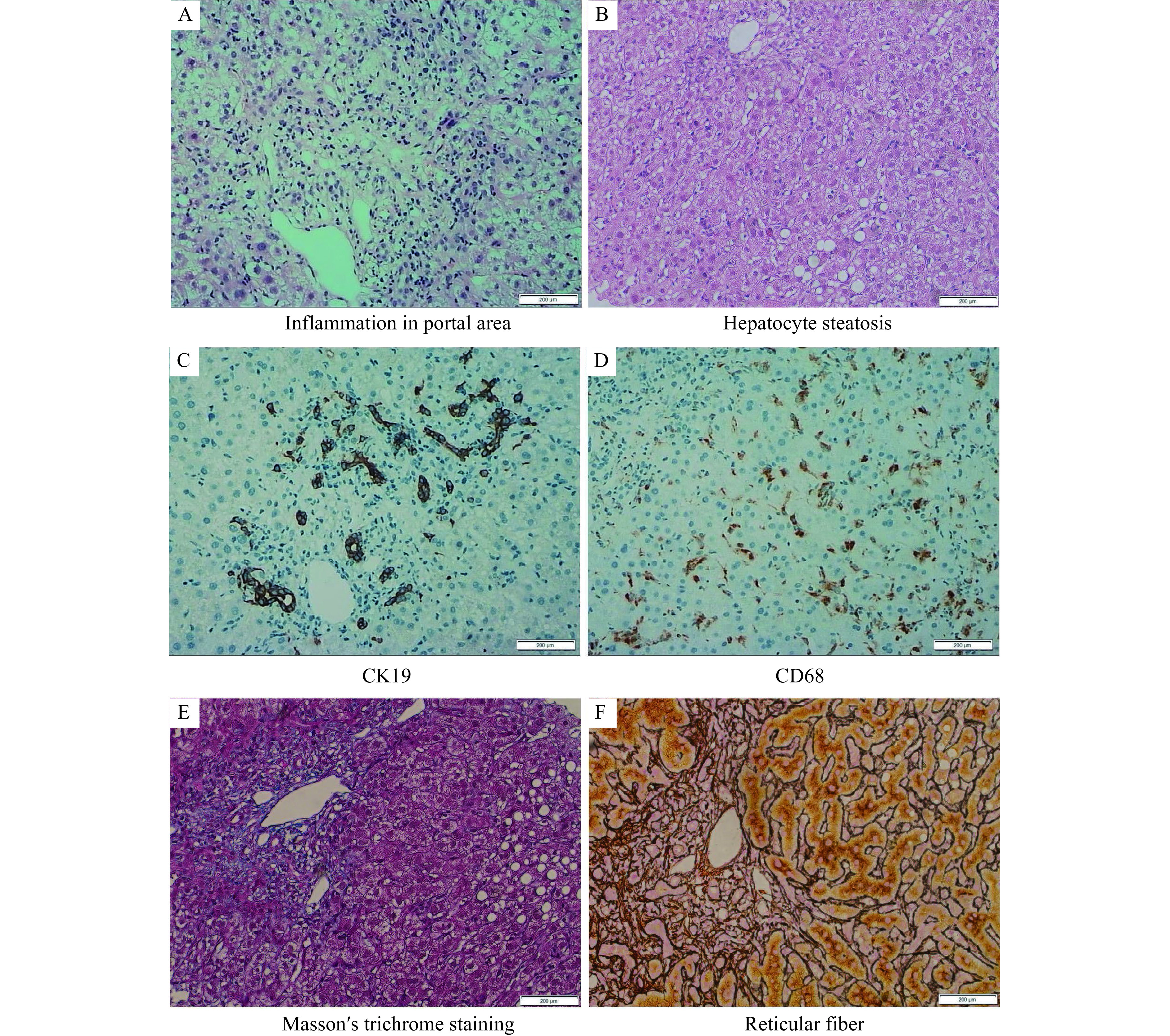
Results of histopathologic examination of liver.

**Figure 3:**
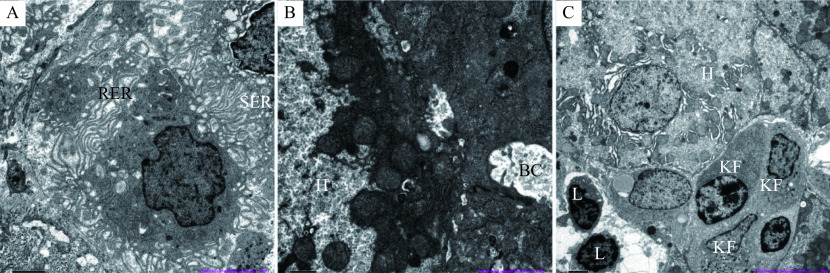
Results of electron microscope examination of liver.

**Figure 4:**
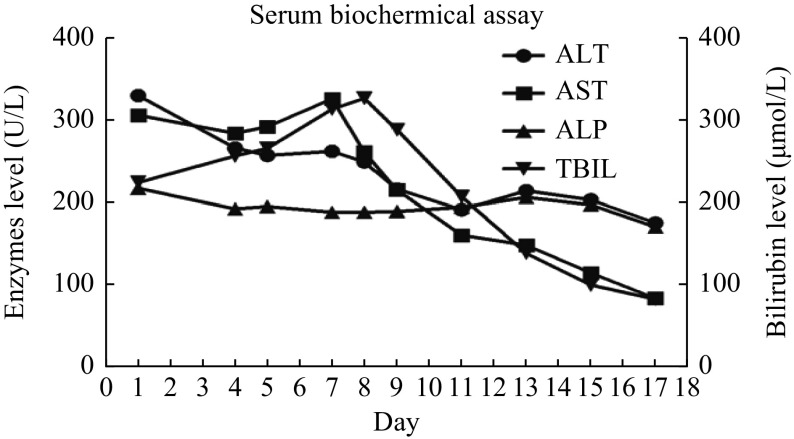
Schematic representation of trends of serum liver function tests during the clinical course and follow-up

To determine the exact cause of the liver injury, we performed detailed dietary survey, requiring the patient to record all her food intake, as well as a liver biopsy. Almost all the foods in the patient's food list belong to the category of regular diet. Then, we required the patient to carefully recall what she had taken before the onset in May. Finally, we focused on a food supplement, *S. versicolor* gum, which was used to treat her obstipation. The *S. versicolor* gum had been used before both of her hospitalization. Her RUCAM score was 7 (***Table 3***), which was consistent with probable DILI. Meanwhile, the histological examination of the liver tissue showed that drug-induced liver injury was considered (***Fig. 2*** and ***3***). Bicyclol tablet, ursodeoxycholic acid capsules, glutathione injection (YaoPharma, China), and ademetionine 1,4-butanedisulfonate injection were administered. Meanwhile, methylprednisolone at a dosage of 60 mg QD was administered for 3 days and sequentially with prednisone at a dosage of 50 mg QD to alleviate liver inflammation, because of the increased level of TBIL (peaking at 326.7 μmol/L; DBIL 263.6 μmol/L). The TBIL decreased obviously after 15 days (***Fig. 4***) and finally returned to normal level after two months with oral medicines; so did other indicators.

**Table 3:** Patient's score on the Roussel Udaf Causality Assessment Method scale at her second admission

Liver injury type	Hepatocellular	Score
Time of onset of the event	Re-exposure	
Time from drug intake until reaction onset	1–15 days (rechallenge)	+2
Course of ALT after cessation of the drug/herb Percentage difference between ALT peak and ULN	Decrease more than 50% within 30 days	+2
Risk factors	No alcohol use/Age< 55 years	0
Concomitant drug/herb	Concomitant drug/herb with incompatible time to onset	0
Search for alternative causes	The 6 causes of groupⅠ ruled out	0
Previous hepatotoxicity of the drug/herb	Reaction unknown	0
Response to unintentional re-exposure	Doubling of ALT with the drug/herb alone, provided ALT below 5×ULN before re-exposure	+3
Total score	Probable	7
ALT: alanine transaminase;ULN: upper limit of normal.			

## Discussion

*S. versicolor* gum is an exudate of *S. versicolor*. Chemically, *S. versicolor* gum is a glycanorhamnogalactouran, with alternating backbone units of α-D-galactouronic acid linked at C4 to α-L-rhamnose at the C2 position. Substitution occurs on the hydroxyl groups by D-galactose and D-glucuronic acid. The exudate can be employed in the food and textile industries as a stabilizer and adhesive.

*S. versicolor* gum is also used as a dietary fiber supplement in its main producing areas, such as Burma, Vietnam, India, and other tropical and subtropical countries. The exudate is allowed to dry on the tree and later collected, broken, cleaned, and sorted. Among the naturally occurring gums, gum karaya from*Sterculia uren*s is profusely used as a food additive. The side effects of *S. versicolor* gum include flatulence and abdominal cramping. A case of esophageal obstruction after ingestion of *Sterculia* has been reported in a 91-year-old man after taking a tablespoonful of *Sterculia* granules (Normacol) without water^[6]^. So far, no case of *S. versicolor*-induced liver damage has been reported. Taking *S. versicolor* gum has become prevalent in southwest of China.

In our case, the differential diagnosis includes hyperthyroidism-induced liver injury as well as fatty liver. We exclude hyperthyroidism-induced liver injury according to the following reasons. The patient had a clear history of hypothyroidism and took levothyroxine tablets regularly. The indicators at the first admission were consistent with hyperthyroidism, which is considered to be medication-induced hyperthyroidism. And the thyroid function almost returned to normal level at the second admission, but obvious abnormal liver injury still existed. Therefore, the possibility of DILI caused by the re-exposure to liver damage drugs was considered, and liver injury caused by hyperthyroidism was no longer considered. Furthermore, thyroid antibodies were positive at the first hospitalization but negative at the second admission, which indicates an active autoimmune situation, as were ANA and ANCA.

Also, the diagnosis of non-alcoholic steatohepatitis should be considered in this case considering the results of abdominal ultrasound and magnetic resonance testing. But this diagnosis was finally excluded by the pathology results that the Steatotic hepatocytes were less than 5%^[7]^.

The causes of liver injury in DILI cases should consider not only the patients' underlying diseases and corresponding therapeutic drugs, but also the medication and diet habits, and the latter may be easily ignored by patients. Apart from epidemiological investigation, DILI of different types also have their own characteristics. For example, DILI due to antibiotics, particularly fluoroquinolones, tends to have a shorter latency period^[8]^, and AST level is usually higher than ALT level in alcohol-induced liver injury^[9]^.

The first and the most important treatment strategy for DILI is timely withdrawal of the suspected liver-injuring drugs^[1]^. Approximately 95% of DILI cases can achieve spontaneous improvement and recover completely once the identified drugs are withdrawn immediately^[1]^. Elevated liver biochemical indices (ALT>3 times and/or bilirubin >2 times the upper limit of normal) are considered to be a predictive factor of severe DILI. In our case, a mortality of approximately 10% was expected^[10]^.

Since the mechanism of *Sterculia*-induced DILI is unclear, more research should be carried out to determine whether *Sterculia* can cause DILI and whether some populations are more sensitive to it than others.
